# Molecular Identification of Bacteria in Tracheal Aspirate Fluid from Mechanically Ventilated Preterm Infants

**DOI:** 10.1371/journal.pone.0025959

**Published:** 2011-10-10

**Authors:** Peter M. Mourani, J. Kirk Harris, Marci K. Sontag, Charles E. Robertson, Steven H. Abman

**Affiliations:** 1 Pediatric Heart Lung Center, Department of Pediatrics, University of Colorado Denver, School of Medicine, Aurora, Colorado, United States of America; 2 Department of Epidemiology, Colorado School of Public Health, University of Colorado Denver, Aurora, Colorado, United States of America; 3 Department of Molecular, Cellular and Developmental Biology, University of Colorado, Boulder, Colorado, United States of America; University of Florida, United States of America

## Abstract

**Background:**

Despite strong evidence linking infections to the pathogenesis of bronchopulmonary dysplasia (BPD), limitations of bacterial culture methods have precluded systematic studies of airway organisms relative to disease outcomes. Application of molecular bacterial identification strategies may provide new insight into the role of bacterial acquisition in the airways of preterm infants at risk for BPD.

**Methods:**

Serial (within 72 hours, 7, 14, and 21 days of life) tracheal aspirate samples were collected from 10 preterm infants with gestational age ≤34 weeks at birth, and birth weight of 500–1250 g who required mechanical ventilation for at least 21 days. Samples were analyzed by quantitative real time PCR assays for total bacterial load and by pyrosequencing for bacterial identification.

**Results:**

Subjects were diagnosed with mild (1), moderate (3), or severe (5) BPD. One patient died prior to determination of disease severity. 107,487 sequences were analyzed, with mean of 3,359 (range 1,724–4,915) per sample. 2 of 10 samples collected <72 hours of life contained adequate bacterial DNA for successful sequence analysis, one of which was from a subject exposed to chorioamnionitis. All other samples exhibited bacterial loads >70copies/reaction. 72 organisms were observed in total. Seven organisms represented the dominant organism (>50% of total sequences) in 31/32 samples with positive sequences. A dominant organism represented>90% of total sequences in 13 samples. *Staphylococcus*, *Ureaplasmaparvum*, and *Ureaplasmaurealyticum* were the most frequently identified dominant organisms, but *Pseudomonas*, *Enterococcus*, and *Escherichia* were also identified.

**Conclusions:**

Early bacterial colonization with diverse species occursafter the first 3 days of life in the airways of intubated preterm infants, and can be characterized by bacterial load and marked species diversity. Molecular identification of bacteria in the lower airways of preterm infants has the potential to yield further insight into the pathogenesis of BPD.

## Introduction

Bronchopulmonary dysplasia (BPD) was initially attributed to mechanical ventilation and oxygen induced lung injury in infants born preterm, as first described by Northway and colleagues in 1967 [1]. Inflammation of the developing lung from these interventions and other causes is a predominate theory underlying thepathogenesisof BPD [2], [3]. Both prenatal and postnatal sources of inflammation, including infection, may directly disrupt lung growth [4] and predispose infants to BPD [5], [6]. Because fetal development takes place in a normally bacteria-free environment [7], premature exposure and colonization of the neonatal respiratory tract by bacteria may impact the neonatal immune response, increase the risk for early and late infections, and contribute to the subsequent development of BPD. Recent studies have shown that airway colonization with specific organisms during the neonatal period was associated with persistent wheeze in childhood [8], suggesting that early specific bacterial exposure and/or the host response contribute to future disease. Yet, little is known about the timing of bacterial exposure and acquisition in the airways of preterm infants, mechanisms that promote or impede colonization of specific organisms, or the inflammatory responses to colonizing bacteria in relation to injury to the developing lung.

Recently developed molecular methods have suggested that the lungs of healthy older children and adults are rich with microbes, which has led to a new understanding of airway colonization and has challenged the traditional theory that human lower airways are sterile [9], [10]. These methods further suggest that traditional culture methods are not adequate to investigate the contribution of microbial communities to disease. In addition to common bacterial organisms that can be readily cultured, there has been clear recognition of the potential pathological impact of other bacteria, such as *Ureaplasma*, *Mycoplasma*, and *Chlamydia*, as causes of perinatal infection that may increase the risk for the subsequent development of BPD [11], [12], [13], [14]. Thus, traditional culture methods are likely not sensitive enough to provide meaningful information on many potential pathological organisms that can affect outcomes in preterm neonates. Culture-independent molecular techniques offer the opportunity to identify the full spectrum of bacteria in biological samples, and these methods may provide new insight into the role of bacterial acquisition in the respiratory tract of newborn preterm infants and the development of BPD. Molecular bacterial identification methods can detail the total bacterial load in a sample, the diversity of bacteria present, and relative abundance of specific bacteria based on the relative proportion of sequences in a sample.

Thus, we hypothesized that early colonization with diverse microbial agents occurs early after birth in ventilated preterm infants, and that application of molecular bacterial identification strategies to serial airway samples can define changes in the microbiome in infants at risk for chronic lung disease. In order to investigate the airway microbiome in patients at high risk for BPD, we performed molecular identification of bacteria in serial tracheal aspirate samples from intubated preterm infants by isolating rRNA genes harvested by PCR to determine the composition and the timing of acquisition of bacterial communities in the lower airways. We compared these results to those obtained by clinically indicated standard laboratory cultures and described the timing of antibiotic administration in relation to the identification of airway bacterial communities.

## Methods

### Study Population

All data and specimens were obtained as part of a prospective observational research protocol, approved by the Colorado Multiple Institutional Review Board. Written informed consent was received from the parents/guardians of all participants. Assent was not obtained because all participants were infants. Criteria for enrollment included a gestational ageof 34 weeks or less, birth within the previous 72 hours, and a birth weight of 500 to 1250 g. To be included in this study analysis, infants also needed to be intubated at enrollment and remain intubated for at least 21 days with successful collection of tracheal aspirate samples at each of 4 times points (within 72 hours of birth, and 7, 14, and 21 days of life). Exclusion criteria werelethal congenital anomalies, complex congenital heart disease (includingan atrial septal defect larger than 1 cm and a ventricular septaldefect larger than 2 mm), or anticipated death prior to hospital discharge. Maternal medical history including complications of pregnancy and birth, and subject data, including clinically obtained respiratory tract cultures and antibiotic administration from the NICU course were collected as part of the prospective study. BPD status was assessed at 36 weeks post conceptual age via head hood oxygen reduction test as previously described [15], anddisease severity categorieswere adjusted for altitude in Denver, Colorado (1600 m).

### Specimen Collection and processing

Tracheal aspirates were collected by instillation of 0.5 mlof sterile 0.9% saline (without preservative) via the endotracheal tube (ETT) and suction applied to a sterile catheter inserted to 0.5 cm below the tip of the ETTas the catheter was slowly withdrawn. The suction catheter was cleared of retained secretions by aspirating an additional 1 ml of the sterile saline. Specimens were immediately spun at 250× g for 20 minutes at °C. The supernatant was removed and the remaining pellet was resuspended with 0.2 mlnormal saline and stored at −70°C.

### DNA Extraction, PCR and Sequencing

DNA was prepared from each sample using the Qiagen EZ1 Advanced platform. 200 µl of clinical sample was extracted using the bacterial card and tissue kit per manufacturer's instructions. The bacterial load of each sample was estimated using a TaqMan quantitative PCR (qPCR) assay described by Nadkarni [16]. Each DNA sample was assayed in triplicate, and assays with coefficient of variation (standard deviation/mean) greater than 20% were repeated. We have validated the reproducibility of this assay in comparable airway samples from children with cystic fibrosis [17]. Copy number was established using a standard curve assembled from 10-fold serial dilutions of plasmid DNA containing a cloned rRNA gene.

DNA sequencing was performed using barcoded primers (27F–338R) compatible with the Roche 454 pyrosequenceras previously reported [18]. Each DNA was amplified in triplicate PCR assays, which were pooled prior to assessing the product by agarose gel electrophoresis. A negative control was run for each barcode in parallel. Any assays where the negative control was positive were repeated. Amplicons of the correct length were arrayed in 96-well format for normalization. Amplicon concentration was normalized using the SequalPrep normalization plate (Invitrogen) prior to mixing [19]. Equal volumes were mixed, concentrated by evaporation and gel purified using the Montage Gel Purification Kit (Millipore). The resulting amplicon pool was sequenced per manufacturer's instructions using the Roche Genome Sequencer FLX system at the Consortium for Comparative Genomics sequencing facility at the University of Colorado Denver.

### Sequence analysis

Sequence data (fasta and quality files) were assigned to samples by bar code and screened for low level quality defects (short sequences <150 nt in length, >1 sequence ambiguity, best read with quality ≥20 over a 10 nt moving window) by the software program BARTAB [20]. Non-bacterial sequences were removed from the dataset by requiring a close match with a bacterial secondary structure rRNA model within Infernal [21]. Sequences identified as potential chimeras by ChimeraSlayer [22] were also removed from the dataset. Taxonomic assignment was done by the Ribosomal Database Project Classifier software [23]. We used a BLAST database containing bacterial isolates identified in the Silva ARB database (version 104) to assign species names [24]. The taxonomic information was used to construct sequence groups with identical taxonomic rank, which were used to calculate ecology statistics for each sample. Shannon diversity and Chao_1_ were computed, using rarefaction (100 replicates) to compensate for differences in number of sequences per sample, using the biodiv functionality contained within the XplorSeq Toolkit [25], [26].

### Statistical analysis

Data were presented graphically, and descriptive statistics were calculated using SAS v 9.2 (Cary, NC). The percent of total sequence count was calculated for each individual organism. Multilevel plots were constructed, displaying the percent of total sequences, clinical culture results, and antibiotic courses for each infant. Bacterial load and Shannon diversity index were plotted individually for each patient. Formal statistical testing was not performed.

## Results

**Figure 1:**
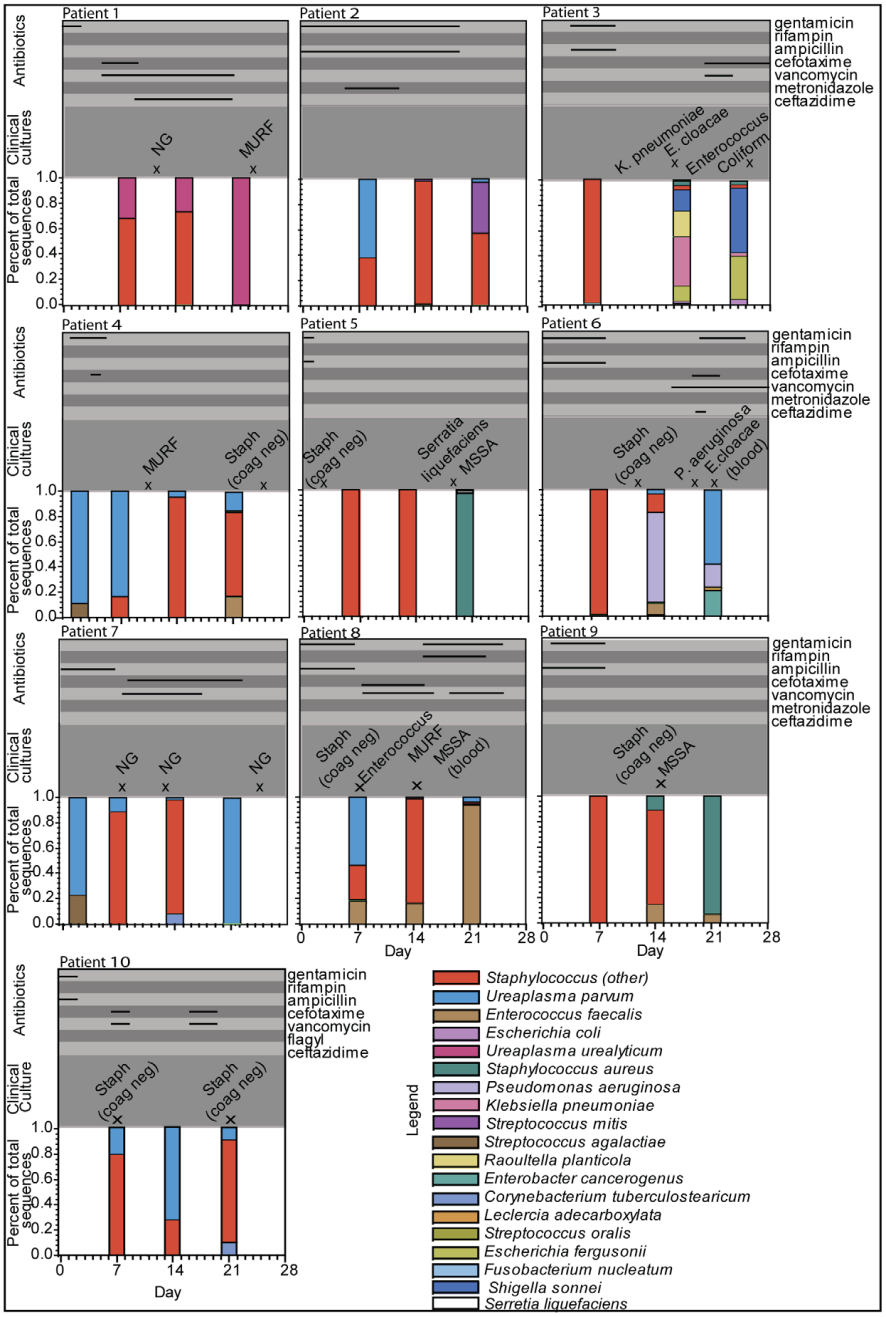
Bacterial Composition of Tracheal Aspirate Samples for Each Patient by Day of Collection. Bacterial composition of each sample is displayed in the bar graphs as a percent of total sequences in the sample. Each species can be identified by color according to the legend. Days of antibiotic administration are displayed on the top of each graph with the solid lines depictingthe duration of specific antibiotic use as noted on right side of the figure. Results of clinically obtained cultures are displayed in the middle section of each graph with an “x” depicting the day they were obtained. All cultures were from tracheal aspirates unless otherwise noted. (NG: no growth, MURF: mixed upper respiratory flora, E. cloacae: *Enterobacter* cloacae, K. pneumoniae: *Klebsiella* pneumonia, MSSA: methicillin sensitive *Staphylococcal* aureus, P. aeruginosa: *Pseudomonas* aeruginosa.

Of 49 subjects enrolled in the larger observational cohort at the time of analysis, 10 patients met the ventilation and sampling criteria for inclusion in this study. Clinical characteristics of the 10 patients are listed in Table 1. Patient 1 died at day of life 40 from methicillin-resistant *Staphylococcus aureus* sepsis; this patient did not have an autopsy performed. The remaining patients were all diagnosed with BPD, ranging from mild to severe. All patients received broad spectrum antibiotics, empirically or for confirmed infection, at some point during the first 21 days of life (Figure 1).

**Table 1:** Patient Characteristics.

	Patient									
	1	2	3	4	5	6	7	8	9	10
**Gender**	Male	Male	Female	Female	Female	Male	Male	Female	Female	Male
**Race**	NHB	NHB	NHW	HW	HW	HW	NHW	NHW	HW	NHW
**Gestational age (PMA, weeks)**	26	27	26	24	25	25	26	26	24	25
**Birth weight (grams)**	776	835	852	605	1000	700	945	770	669	905
**APGAR 1 min.**	3	1	1	3	1	1	6	3	3	5
**APGAR 5 min.**	8	4	1	6	5	1	5	7	3	7
**BPD Severity**	*Expired*	Severe	Mild	Severe	Mild	Severe	Mod.	Severe	Mod.	Severe
**Mechanical Ventilation Days**	39	42	49	59	22	33	44	46	27	33
**CPAP days**	0	26	0	15	14	17	11	12	6	21
**Oxygen days (including positive pressure support)**	39	131	98	120	83	112	112	153	126	128
**Discharged on oxygen**	Yes	No	No	Yes	Yes	No	Yes	Yes	Yes	Yes
**NICU days**	39*	142	116	120	83	128	112	153	126	128
**Gestational age at time of discharge, weeks**	31.6*	47.3	42.6	41.1	36.7	43.3	41.9	47.7	41.9	43.3

NHB: Non-Hispanic Black, NHW: Non-Hispanic White, HW: Hispanic White, PMA: post mestrual age, BPD: bromchopulmonary dysplasia, CPAP: continuous positive airway pressure, NICU: neonatal intensive care unit,

*Expired.

**Figure 2:**
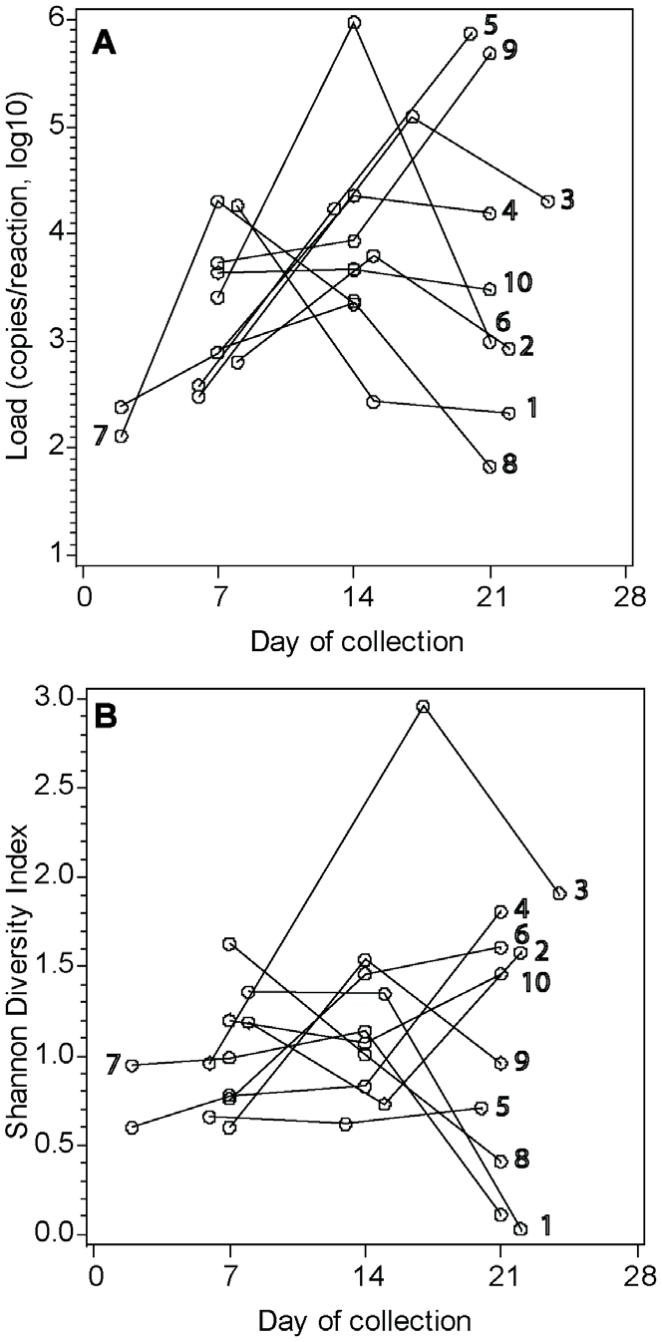
Comparison of Bacterial Load and Shannon Diversity Index by Age. **A.** Distribution of total bacterial loads by sample. Lines connect samples from each patient, labeled with the patient number corresponding to clinical data presented in Table 1. **B.** Diversity of organisms by sample as determined by the Shannon Index. Lines connect samples from each patient, labeled with the patient number corresponding to clinical data presented in Table 1.

The average yield of DNA from each sample was 32.9 ng/µl (range: 0.4–143.6). A total of 107,487 sequences were analyzed, with an average number of 3,359 (range: 1,724–4,915) per sample. Only 4 of 10 samples collected <72 hours of life had detectable bacterial signal by qPCR to assess bacterial load. Further, only 2 of these samples contained adequate load for successful sequence analysis of the bacterial community. All samples collected from day 7–21 of life contained detectable bacterial load (>70 copies/reaction), and each sample was successfully amplified for bacterial identification. The distribution of bacterial loads by day of collection for each subject is shown in Figure 2A. Seventy-two organisms were observed in total. The average number of organisms identified per sample was 7 (range 2–23). Sequences observed ≥1.0% of total in a sample represent 19organisms (96.5% of total sequences; 1–9 organisms per sample). Chao_1_, a nonparametric estimate of actual species richness in a sample, predicted on average, 8 organisms in each sample (range 1–46). The diversity of organisms in samples, determined by the Shannon Index, is displayed by day of collection for each subject in Figure 2B.

A dominant organism, defined as an organism representing >50% of total sequences was identified in31 of 32 samples with positive sequences(7 organisms represented). A dominant organism represented >90% of total sequences in 13 samples (6 organisms). Distributions of bacterial sequences in the samples for each patient are displayed in Figure 1 along with results of clinically obtained cultures and the timing of antibiotic administration. *Ureaplasmaparvum* was the dominant organism in the two positive samples from <72 hours, one of which was from a subject exposed to chorioamnionitis (subject 4). *Ureaplasmaparvum* or *Ureaplasmaurealyticum* represented the dominant organism in 9 samples from 6 subjects, 5 samples of which were from subjects without evidence of *Ureaplasma* on the initial sample. *Staphylococcus* species represented the dominant organism in 19 samples (17 were coagulase negative *Staphylococcus*). Of these, *Staphylococcus* represented>90% of sequences in7 samples. *Staphylococcus aureus* was the dominant species in 2 samples (both collected on day 21). *Pseudomonasaeruginosa* (1 sample; day 14), *Enterococcus faecalis* (1 sample; day 21), and *Escherichia coli* (1 sample; day 21) were the other dominant organisms identified in samples.

Based on suspected lung infection by the physicians managing these patients during the course of the study, 19 additional tracheal aspirate samples were obtained for bacterial culture within at least 72 hours of research tracheal aspirate collections (Figure 1). Of these, 14 (74%) had positive bacterial growth, with specific organisms identified in 11 samples and “mixed upper respiratory flora (MURF)” reported in the remaining 3 samples. Sequencing identified all bacteria that grew in the clinically indicated cultures (n = 11 positive cultures). Six of these 11 cultures grew 2 organisms, however, the bacteria identified by culture did not always represent the organisms with the most sequences identified by molecular methods (n = 5 cultures). Non-cultivatable bacteria, such as *Ureaplasma*, were a significant source of discrepancies between the molecular strategies versus standard culture approaches.

Potential pathogens identified by sequencing that were not identified by culture include *Ureaplasma* (3 cultures), *Pseudomonas* (1 culture), and *E. coli* (1culture). Of the 5 samples that were obtained for clinical indications that had no bacterial growth, the paired research samples revealed *Staphylococcus* species as the dominant organism in 3 samples, *Ureaplasma* species as the dominant organism in one sample, and no bacterial amplification in the remaining sample. Systemic antibiotics were administered for some period of time during the first 21 days of life in all patients. The median duration of antibiotic administration was 12 days (range, 2–21). The median duration on 2 or more antibiotics was 10 days (range, 2–19). All samples collected after 72 hours of life that had *Ureaplasma* species as the dominant organism (n = 7) had been treated with 2 or more antibiotics in the interval between research samples.

## Discussion

We performed molecular identification of bacteria in serial tracheal aspirate samples from intubated preterm infants by isolating rRNA genes harvested by PCR to determine the composition of bacterial communities and the timing of acquisition in the airways of these infants. We found that during the first 72 hours after birth, the majority of tracheal aspirate samples had low or undetectable bacterial loads. However, within 7 days of life, sustained bacterial colonization with diverse species is present in the airways of intubated preterm infants. 96.9% of samples with bacterial detection exhibited a dominant organism, with *Staphylococcus* and *Ureaplasma* species most frequently identified as the dominant organisms. Molecular methods identified all species detected by traditional clinical cultures, but also identified potential pathogens, including *Ureaplasma*, that were not detected by standard bacterial culture methods. Thus, improved resolution of bacterial detection with regard to overall bacterial load, species diversity within the microbiome or the identification of specific organisms for directed therapy, has the potential to yield further insights into the pathogenesis of BPD and late respiratory outcomes of preterm newborns.

These findings are important because early respiratory infection and inflammation have been shown to impair alveolar development [27] and contribute to the development of BPD [28]. Inflammatory injury disrupts lung growth in part by production of pro-inflammatory cytokines [29]. Although pro-inflammatory cytokines have been associated with the development of BPD and adverse outcomes [30], [31], [32], variable assessments of the pro-inflammatory response, and lack of context specificity (identification of all aspects of the perinatal milieu contributing to disease) have precluded the use of cytokines as adequate biomarkers for BPD. While antenatal and postnatal infections, hyperoxia, and mechanical ventilation have garnered much attention as inflammation provoking stimuli, the possible impact of airway colonization on the development of BPD is less clear and has generated contradictory results [33]. The presence of *Ureaplasma* in the respiratory tract of preterm infants, even without signs of infection, has been associated with a pro-inflammatory response and an increased risk for BPD [34]. Although earlier trials of erythromycin treatment for the treatment of *Ureaplasma* in preterm infants failed to show benefit [35], [36], [37], [38], a recent trial of azithromycin treatment revealed a significant reduction in the endpoint of BPD or death in the subgroup of patients who were found to be colonized or infected with *Ureaplasma* [39]. Thus, Ureaplasma remains a plausible but still unconfirmed contributor to BPD. *Ureaplasma* was the dominant organism in at least one sample for 6 of the 10 infants in our study. *Ureaplasma* was found to be the dominant organism in late samples even when not detected in initial samples, suggesting the possibility of post-natal acquisition and/or the presence of environmental factors, such as antibiotic administration, that encourage selection of this genus. The association between antibiotic administration, *Ureaplasma* colonization, and risk for BPD should be explored further in future studies. Exposure to colonizing bacteria may impact the preterm infant's immune response and subsequent lung development. Improved resolution of bacterial detection techniques as demonstrated here in conjunction with assessments of inflammation could improve our understanding of the developing host immune response in premature infants and its relationship to pathogen exposure.

Molecular methods have contradicted the traditional theory that human lower airways are sterile by demonstrating that the lungs of healthy older children and adults are rich with microbial flora [9], [10], and that traditional culture methods are not adequate to investigate the contribution of microbial communities to disease. Our results, together with a previous report [40] suggest that microbial communities are present early in the lower airways of intubated preterm infants. Rapidly accumulating evidence demonstrates that microbial communities play an important role in human physiology [41], [42]. Several diseases have been associated with changes in composition and diversity of these microbial communities [43], [44], [45]. Some have shown that microbiota of limited diversity and relative absence of commensal organisms are associated with increased inflammation, barrier permeability, and disease status [46], [47]. Early evaluations of the respiratory tract microbiota in pulmonary diseases such as cystic fibrosis, asthma, and COPD [9], [10] also reveal shifts in microbial composition compared to healthy patients, suggesting the composition of the respiratory tract microbiota can play a direct role in pathophysiology of lung disease. Neonatal oropharyngeal colonization with *Streptococcus pneumoniae*, *Moraxella catarrhalis*, *Haemophilusinfluenzae*, or a combination of these organisms, but not colonization with *Staphylococcusaureus*, was significantly associated with persistent wheeze in childhood [8]. Children living on farms are exposed to a wider range of microbes than were children living in urban environments, and these children appear relatively protected from asthma and atopy [48]. Together, these studies suggest the respiratory tract microbiota early in life can also modulate the host immune response and future lung disease.

Only 2of 10 samples collected within 72 hours after birth exhibited sufficient bacterial load for sequence analysis, suggesting the lower airways of preterm infants typically have comparatively low bacterial loads. One of these two samples was obtained from an infant exposed to chorioamnionitis and revealed *Ureaplasmaparvum*, a known cause of chorioamnionitis, as the dominant organism. Although a diverse array of bacteria were detected within the samples of these 10 infants, the diversity of organisms within samples was far less than detected in respiratory samples of older children and adults [9], [10]. Further, bacterial diversity was not associated with age of the infant or BPD severity.

The use of antibiotics administered to treat confirmed or potential infections may have long term effects on the composition of the airway microbial community. Potential negative consequences include the elimination of commensal bacteria and selection for other pathogenic bacteria, especially those that are not readily detected by clinical culture techniques and that promote chronic airway inflammation. Further studies will need to investigate the potential role of antibiotics on the composition of the airway microbiota and the impact on early lung development.

There are several limitations to our study. First, the small sample size in this pilot study precludes rigorous evaluation of the presence of specific bacteria, bacterial loads, or species diversity to the risk of BPD. All of the infants included in this study were less than 28 weeks gestation and required mechanical ventilation for at least 21 days, which represents a very high risk population for severe BPD. Serial evaluation of the airway microbiome in preterm infants who do not develop BPD will be important to determine how the composition of the airway microbiome is related to risk of BPD. Since preterm infants who do not develop BPD often do not require long periods of mechanical ventilation support, noninvasive methods of airway sampling will be required. Second, all infants in this study were treated with systemic antibiotics which are likely to impact the evolution of microbial composition, limiting the ability to ascertain the natural acquisition of airway colonization after birth. The presence of endotracheal tubes also impacts the access of microbes to lower airways. Although we attempted to compare molecular detection methods to clinical culture results, the samples were not simultaneously obtained. Therefore, the discrepancies between the two results cannot be accounted for by methodology alone. In addition, sampling of tracheal aspirates may not adequately reflect bacterial exposure at the alveolar level. Simultaneous sampling of the upper airway, lower airways and alveolar samples are required to provide a more complete assessment of bacterial colonization. Finally, future studies are needed to more directly link the presence of specific airway organisms, airway bacterial load, and diversity of the microbiota with altered innate immunity and inflammatory cytokines to better understand how early colonization impacts lung inflammation.

In summary, early bacterial colonization with diverse species are present in the airways of intubated preterm infants, and can be characterized by bacterial load and species diversity. Molecular detection of *Ureaplasma* species in the airways of preterm infants late in the course of mechanical ventilation provides further support for the concept that *Ureaplasma* may be involved in the pathogenesis of BPD and requires further investigation. Molecular identification of bacteria in the lower airways of preterm infants has the potential to yield further insight into the pathogenesis of BPD and to identify novel therapeutic targets.
